# Hemispheric Asymmetry of Auditory Mismatch Negativity Elicited by Spectral and Temporal Deviants: A Magnetoencephalographic Study

**DOI:** 10.1007/s10548-013-0347-1

**Published:** 2013-12-24

**Authors:** Hidehiko Okamoto, Ryusuke Kakigi

**Affiliations:** Department of Integrative Physiology, National Institute for Physiological Sciences, 38 Nishigo-Naka, Myodaiji, Okazaki, 444-8585 Japan

**Keywords:** Auditory evoked response, Hemispheric laterality, Magnetoencephalography (MEG), Mismatch negativity (MMNm)

## Abstract

One of the major challenges in human brain science is the functional hemispheric asymmetry of auditory processing. Behavioral and neurophysiological studies have demonstrated that speech processing is dominantly handled in the left hemisphere, whereas music processing dominantly occurs in the right. Using magnetoencephalography, we measured the auditory mismatch negativity elicited by band-pass filtered click-trains, which deviated from frequently presented standard sound signals in a spectral or temporal domain. The results showed that spectral and temporal deviants were dominantly processed in the right and left hemispheres, respectively. Hemispheric asymmetry was not limited to high-level cognitive processes, but also originated from the pre-attentive neural processing stage represented by mismatch negativity.

**Electronic supplementary material:**

The online version of this article (doi:10.1007/s10548-013-0347-1) contains supplementary material, which is available to authorized users.

## Introduction

Functional hemispheric asymmetry in the human brain has been investigated since the late nineteenth century (Wernicke 1874; Broca 1861). In addition to the classical behavioral observations of neurological disorder patients, recent neuroimaging techniques have made it possible to investigate conscious healthy human brains, and have revealed left hemispheric dominance for speech processing and right hemispheric dominance for music processing (Zatorre et al. 1994, 2002; Griffiths et al. 1999; Belin et al. 2000; Eulitz et al. 1995; Szymanski et al. 2001; Alho et al. 1998). However, functional hemispheric asymmetry in the human brain may not be limited to high-level cognitive neural processes, but may start from the lower neural processing level of basic acoustic features (e.g. frequency, interval, duration, and intensity).

Natural sounds have specific spectral distributions that change over time according to specific temporal sequences. Both spectral and temporal sound features have been shown to play an important role in the perception of natural sounds (Moore 2003); however, the importance of these features appears to differ between sound types, with spectral processing being of particular importance for music perception (Vos and Troost 1989; Warrier and Zatorre 2002) and temporal cues being essential for speech perception (Shannon et al. 1995; Drullman et al. 1994a, b). Recent functional magnetic resonance imaging (Jamison et al. 2006), positron emission tomography (Zatorre and Belin 2001), and magnetoencephalography (MEG) (Okamoto et al. 2009) studies have demonstrated using artificial basic auditory stimuli (e.g. pure tones and pulse-trains) that temporal changes are dominantly processed in the left hemisphere, whereas spectral changes are dominantly processed in the right. The well-known functional human hemispheric asymmetry observed for speech and music processing may not be limited to conscious high-level cognitive processes, but may be at least partially related to the pre-attentive processing of low-level acoustic features.

Mismatch negativity (MMN) and its magnetic counterpart MMNm are auditory evoked components that reflect the cortical pre-attentive discrimination of auditory stimuli as well as auditory memory traces (Näätänen et al. 1978, 2007; Kujala et al. 2007). MMN(m) is elicited by violations of regularities in sound streams and can be recorded without any motor or other response and can even be obtained from inattentive patients and infants. Previous studies have shown that MMN(m) elicited by speech sounds was significantly lateralized to the left hemisphere (Alho et al. 1998), whereas MMN(m) elicited by musical notes was dominantly processed in the right hemisphere (Lappe et al. 2013; Tervaniemi et al. 1999). However, whether the hemispheric asymmetries of the pre-attentive MMN(m) are limited to meaningful auditory stimuli (e.g. speech and music) or originate from the basic spectral and temporal sound features of these sound stimuli remains unknown.

Therefore, the aim of the present study was to investigate the hemispheric laterality of MMNm evoked by spectral versus temporal sound deviants that do not convey specific phonological, grammatical, or musical information. In order to exclude the possibility that the laterality of the MMNm originated from the sound stimulus itself, we counter-balanced total auditory inputs identical between spectral and temporal deviant conditions. The results of the present study provide a new insight into how the left and right hemispheres pre-attentively deal with the spectral and temporal features of natural sound signals.

## Materials and Methods

### Subjects

Thirteen healthy subjects participated in this study (five females; mean ± standard deviation: 32.1 ± 6.2 years). All participants had normal hearing, had no history of psychological or neurological disorders, and were unambiguously right-handed [assessed via the Japanese version of ‘‘Edinburgh Handedness Inventory’’ (Oldfield 1971)]. All participants were fully informed about the study and gave written informed consent for their participation in accordance with the procedures approved by the Ethics Commission of the National Institute for Physiological Sciences, Okazaki, Japan. The study conformed to the Code of Ethics of the World Medical Association (Declaration of Helsinki).

### Stimuli and Experimental Design

**Fig. 1:**
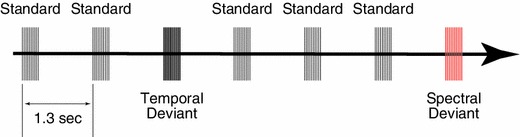
Schematic depiction of the sound stimulation. Standard test stimuli (70 %) were presented together with spectral deviants (SD: 15 %) and temporal deviants (TD: 15 %) within an oddball paradigm

The experimental design is schematically represented in Fig. 1. The test stimulus (TS) was either a 30 Hz (TS30) or 60 Hz (TS60) click-train, which was one-octave band-pass filtered either between 500 and 1,000 Hz (TS30_Low (Supplementary Audio 1S) and TS60_Low (Supplementary Audio 2S)) or between 1,000 and 2,000 Hz [TS30_High (Supplementary Audio 3S) and TS60_High (Supplementary Audio 4S)]. The TS had a duration of 330 ms and the sound onset asynchrony between the TS was 1,300 ms. One of the TS were presented as standard stimuli with 70 % probability pseudo-randomly intermixed with spectral deviants (SD: 15 % probability) and temporal deviants (TD: 15 % probability) in an oddball sequence as demonstrated in Fig. 1. In case of SD, band-pass filter settings changed from the standard stimulus, while the type of the click-train remained identical (standards and SD: TS30_Low and TS30_High, TS30_High and TS30_Low, TS60_Low and TS60_High, TS60_High and TS60_Low). On the other hand, in case of TD, the filter settings remained identical, while the type of click-train changed from the standard sound stimulus (standards and TD: TS30_Low and TS60_Low, TS30_High and TS60_High, TS60_Low and TS30_Low, TS60_High and TS30_High). More than two standard stimuli were presented before a deviant stimulus (SD or TD). Each MEG session consisted of four blocks. Each block contained four sub-blocks that pseudo-randomly adopted TS30_Low, TS30_High, TS60_Low, and TS60_High as the standard TS, respectively. Consequently, all TS types were presented with a probability of 25 % in one block. Each sub-block had 21 SD, 21 TD, and 98 standard stimuli, resulting in a total number of 336 trials for each deviant stimulus and 1,568 trials for the standard condition. All sounds were diotically presented through plastic tubes 1.5 m in length and earpieces fitted to the subject’s ears. Before starting an MEG measurement, each subject’s hearing threshold for TS30_Low was individually determined for each ear. During the MEG recording session, TS30_Low was presented at an intensity of 60 dB above the individual sensation level, and other TS were adjusted to have power identical to TS30_Low. In order to keep the test subjects alert and distracted from the auditory signals, a self-chosen silent movie with captions was presented during the MEG recordings. Questions regarding the content of the movie were asked at the end of the measurement to ensure that the subjects had watched the movie.

### Data Acquisition and Analysis

Auditory evoked fields were recorded with a helmet-shaped, 306-channels MEG system (Vector-view, ELEKTA, Neuromag, Helsinki, Finland) with 102 identical triple sensor elements located in a silent, magnetically shielded room. We analyzed the MEG signals recorded by 204 planar-type gradiometers, detecting the largest signals over the corresponding cerebral sources. Signals were passed through a 0.03–200 Hz band-pass filter and digitized at 600 Hz. The magnetic fields evoked by TS were selectively averaged for each condition (standard, SD, and TD) including pre- and post-stimulus intervals (−100 to 600 ms). In the present study, TS onset (latency = 0 ms) was defined when the first click of the TS reached the eardrum simulated by an artificial ear (Type 4157, Brüel & Kjær Sound and Vibration Measurement, Nærum, Denmark). Subjects were instructed not to move their heads during the recordings and their compliance was monitored through a video camera by the experimenter. In order to improve the signal-to-noise ratio of the auditory evoked magnetic responses, epochs containing amplitude changes greater than 2.7 pT within the pre- and post-stimulus intervals (−100 to 600 ms) were automatically discarded as artifact-contaminated epochs. After artifact rejection, epochs were averaged for each condition (standard, SD, and TD), regardless of the sound types (TS30_Low, TS30_High, TS60_Low, and TS60_High). To analyze the MMNm component, which is elicited by deviant auditory signals (Näätänen et al. 2007; Alho 1995), the averaged auditory evoked fields in each condition (SD, TD, and standard stimuli) were 1–30 Hz band-pass filtered in order to extract the transient evoked responses, and the baseline was corrected relative to the 100 ms pre-stimulus interval. Thereafter, in order to obtain the MMNm waveforms elicited by SD (MMNm_SD) and TD (MMNm_TD), the auditory evoked fields elicited by the standard TS were subtracted from those elicited by SD and TD. The onset of SD matched with the first click of the TS (latency = 0 ms), whereas TD did not occur at the first click of the TS. When 30 Hz click trains (TS30_Low or TS30_High) were used as the standard TS, the temporal deviant occurred at the presentation of the second click of TS60_Low or TS60_High (latency = 16.7 ms). When 60 Hz click trains (TS60_Low or TS60_High) were used as the standard TS, the onset of TD could be the timing of the missing second click of the standard stimuli (latency = 16.7 ms). Therefore, after obtaining the subtracted magnetic waveforms (MMNm_SD and MMNm_TD) the latency of MMNm_TD was offset by a reduction in 16.7 ms and was then used for the subsequent statistical analysis.

In order to investigate differences in the magnetic sensors, the time courses of the root-mean-square (RMS) amplitudes of the subtracted magnetic fields (MMNm_SD or MMNm_TD) were calculated by using all of the left-side (96 sensors) or right-side (96 sensors) planar-type gradiometers in each subject. The most prominent RMS peak in each hemisphere ranging from 100 to 250 ms after the sound onset was defined as the MMNm response in each subject. The mean RMS value within the 10 ms time window around the RMS peak in each condition, each side, and each subject was used in statistical analysis. The mean RMS amplitudes and latencies of the MMNm responses were evaluated separately by means of repeated-measures analyses of variance (ANOVA) using the two factors DEVIANT_CONDITION [spectral deviant (SD) vs. temporal deviant (TD)] and HEMISPHERE (left vs. right).

The estimated single dipole source strength was shown to be modulated easily by the depth of the estimated location (Hillebrand and Barnes 2002). We could obtain reliable source strengths using identical source locations and orientations between conditions. In order to improve the signal-to-noise ratio, we averaged MMNm_SD and MMNm_TD in each subject and used the averaged magnetic waveforms to estimate the single equivalent current dipoles reflecting the MMNm response. The peak MMNm response was initially identified as the maximal RMS value of the global field power between 100 and 250 ms after TS onset. A 10 ms interval around the MMNm peak latency was selected, and the source locations and orientations were estimated using single equivalent current dipole modeling (one dipole per hemisphere) for each subject individually (BESA Research 5.3.7, BESA GmbH, Germany). We calculated the two equivalent current dipoles (one dipole per hemisphere) simultaneously by using all whole-head planar-type gradiometers (204 channels) for the MMNm source estimation. Dipole estimation was not successful in one subject, which reduced the number of subjects to *N* = 12. The goodness-of-fit for the MMNm dipoles of the remaining 12 subjects was more than 80 % (mean ± standard deviation: 89.0 ± 3.0). The estimated sources, which were fixed in location and orientation for each hemisphere of each subject, served as a spatial filter (Tesche et al. 1995) to calculate the source strength for each condition (SD and TD) and in each hemisphere (left and right) of each subject. The mean source strength within the 10 ms time window around the peak MMNm latency was used for further statistical analysis of the MMNm. In order to evaluate the effects of the deviant type and hemisphere, the source strengths and latencies of the estimated equivalent current dipoles corresponding to the MMNm responses elicited by the deviant stimuli (SD and TD) in each hemisphere were evaluated separately via a repeated-measures ANOVA using the two factors DEVIANT_CONDITION (SD vs. TD) and HEMISPHERE (Left vs. Right).

## Results

**Fig. 2:**
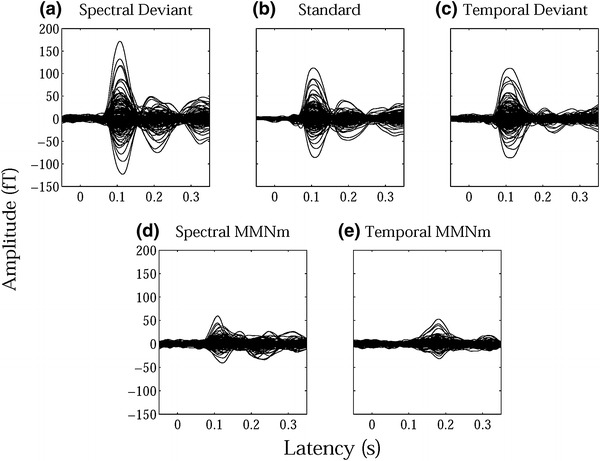
Examples of individual magnetic waveforms. The *upper panels* represent the auditory evoked fields of one representative subject elicited by **a** spectral deviant (SD), **b** standard, and **c** temporal deviant (TD) sound stimuli. The *lower panels* show the magnetic waveforms obtained by the subtraction between **a** and **b** [**d** spectral mismatch negativity (MMNm_SD)] and between **c** and **b** [**e** temporal mismatch negativity (MMNm_TD)]

Twelve subjects (except for one excluded subject) underwent an adequate number of trials to obtain auditory evoked fields for each condition after the artifact rejection [mean ± standard deviation: SD = 332.7 ± 3.6 (99.0 ± 1.1 %), TD = 333.8 ± 1.7 (99.3 ± 0.5 %), standard stimuli = 1556.2 ± 9.3 (99.2 ± 0.6 %)]. An example of individual magnetic field waveforms in each condition (SD, TD, and standard) and subtracted waveforms [MMNm_SD (SD–standard), MMNm_TD (TD–standard)] is shown in Fig. 2, which demonstrates the clear N1m-responses elicited by TS onset in the upper panels as well as MMNm-responses in the subtracted waveforms in the lower panels.

**Fig. 3:**
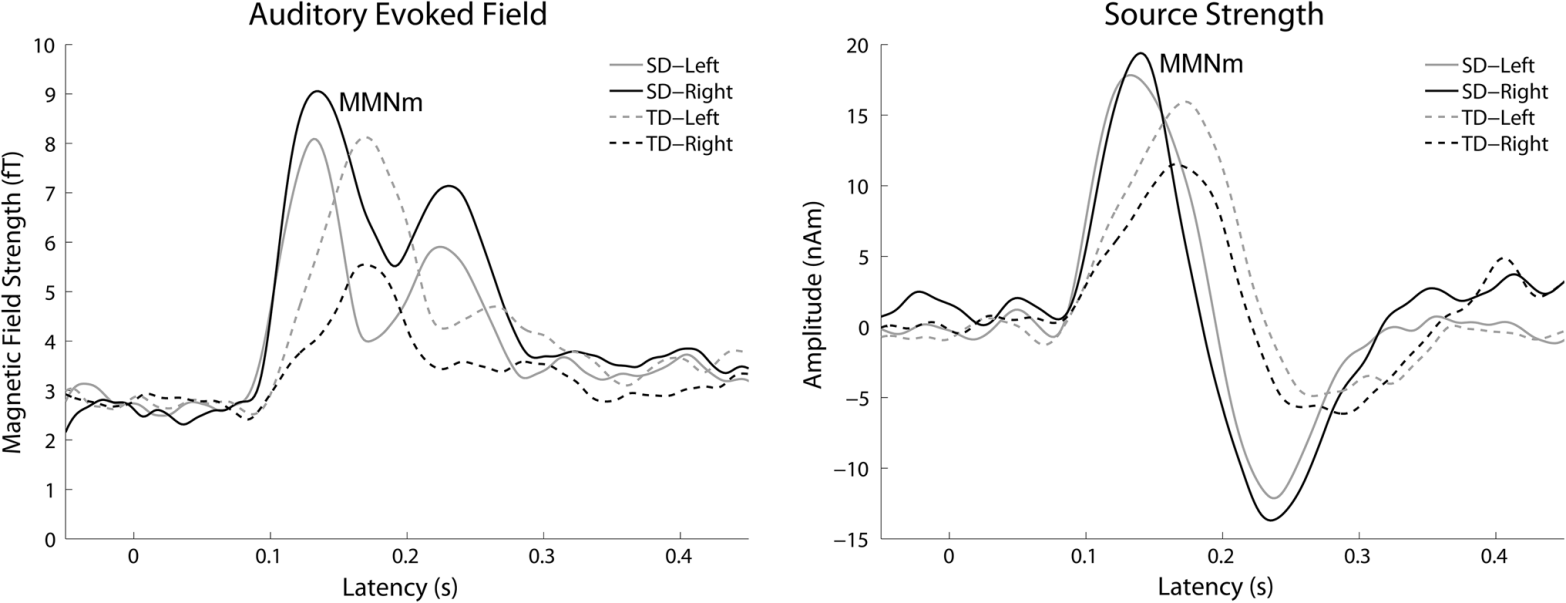
Grand-averaged (*N* = 12) root-mean-square (RMS) values of the magnetic fields (*left panel*) and grand-averaged source strengths (*right panel*) of the mismatch negativity (MMNm) waveforms. *Solid* and *dashed lines* represent the spectral deviant (MMNm_SD) and the temporal deviant (MMNm_TD) conditions, respectively. *Gray lines* represent the* left* sensor (*left panel*) and* left* hemisphere (*right panel*) and *black lines* represent the* right* sensor (*left panel*) and* right* hemisphere (*right panel*)

The calculated means of the RMS values of the auditory evoked fields for each condition (MMNm_SD and MMNm_TD) in each hemisphere averaged across 12 subjects are displayed in Fig. 3, in which the RMS waveforms elicited by TD were shifted 16.7 ms to the left-side in order to adjust the timing of the deviant sound onset. Clear MMNm responses were observed in both conditions and hemispheres. The RMS peaks in the MMNm_TD condition were later than those in the MMNm_SD condition in both hemispheres.

**Fig. 4:**
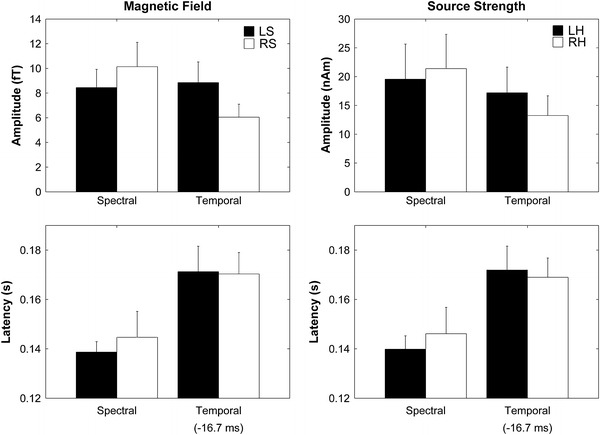
The* left* and* right* graphs display the mean root-mean-square (RMS) values and latencies of the magnetic fields corresponding to the mismatch negativity (MMNm) and mean MMNm source strengths and latencies with *error bars* denoting 95 % confidence intervals, respectively. *Filled bars* denote the left sensor (LS: *left panels*) and left hemisphere (LH: *right panels*) responses and *open bars* denote the right sensor (RS: *left panels*) or right hemisphere (RH: *right panels*) responses

The mean RMS amplitudes and latencies of the MMNm responses averaged across 12 subjects for each condition in each hemisphere are presented in Fig. 4 with error bars denoting the 95 % confidence intervals calculated by the means of bootstrap resampling tests (iteration = 100,000). The repeated-measures ANOVA applied to the maximal RMS amplitudes of the MMNm responses in each hemisphere resulted in a significant main effect for DEVIANT_CONDITION (F_(1,11)_ = 11.78, *p* < 0.01), but not for HEMISPHERE (F_(1,11)_ = 2.53, *p* = 0.14). Additionally, a marginal trend toward significance was observed in the interaction between DEVIANT_CONDITION and HEMISPHERE [F_(1,11)_ = 4.54, *p* = 0.056]. The repeated-measures ANOVA applied to the latencies of the maximal RMS amplitudes of the MMNm responses resulted in a significant main effect for DEVIANT_CONDITION [F_(1,11)_ = 36.30, *p* < 0.001], but neither a significant main effect nor a significant interaction were observed [HEMISPHERE [F_(1,11)_ = 0.33, *p* = 0.58]; DEVIANT_CONDITION × HEMISPHERE [F_(1,11)_ = 0.78, *p* = 0.40].

The calculated means of the MMNm source strength waveforms for each hemisphere averaged across 12 subjects are displayed in Fig. 3, in which MMNm source strength waveforms elicited by TD were shifted 16.7 ms to the left-side. Clear MMNm-responses ranging between 100 and 200 ms were observed in both hemispheres after TS onset. The mean MMNm source strengths and latencies averaged across 12 subjects for each condition in each hemisphere are presented in Fig. 4 with error bars denoting the 95 % confidence intervals calculated by means of bootstrap resampling tests (iteration = 100,000). The repeated-measures ANOVA applied to the MMNm source strengths revealed a significant main effect for DEVIANT_CONDITION [F_(1,11)_ = 6.44, *p* < 0.03]. Additionally, a significant interaction was observed between DEVIANT_CONDITION and HEMISPHERE [F_(1,11)_ = 6.67, *p* < 0.03], which indicated that the MMNm response elicited by SD was relatively larger in the right hemisphere, whereas the MMNm response elicited by TD was relatively larger in the left hemisphere. The repeated-measures ANOVA applied to the MMNm latencies revealed a significant main effect for DEVIANT_CONDITION [F_(1,11)_ = 48.45, *p* < 0.001], but no significant interaction between factors: MMNm_TD was significantly longer than that of MMNm_SD.

We also analyzed MMNm source strengths and latencies when the MMNm_TD was not shifted by 16.7 ms during the calculation. A repeated-measures ANOVA performed on the MMNm source strengths revealed a significant main effect for DEVIANT_CONDITION [F_(1,11)_ = 6.29, *p* < 0.03] and a significant interaction between DEVIANT_CONDITION and HEMISPHERE [F_(1,11)_ = 6.87, *p* < 0.03]. A repeated-measures ANOVA performed on the MMNm latencies revealed a significant main effect for DEVIANT_CONDITION [F_(1,11)_ = 125.3, *p* < 0.001], but no significant interaction between factors.

## Discussion

The results obtained in the present study clearly demonstrated a difference in the hemispheric laterality of MMNm amplitudes between SD and TD conditions. The amplitudes of MMNm evoked by SD (MMNm_SD) were relatively larger in the right, whereas those evoked by TD (MMNm_TD) were relatively larger in the left (Figs. 3, 4). No hemispheric difference was observed in the MMNm latency; however, the latencies of MMNm_TD were significantly longer than those of MMNm_SD in both the left and right hemispheres even when the onset time difference between SD and TD (16.7 ms) was considered. In contrast to previous studies (Alho et al. 1998; Shtyrov et al. 2000), which also investigated the hemispheric asymmetry of MMN(m), the total sound inputs were identical between SD and TD conditions in the present study. Therefore, the sound property itself cannot explain the obtained results; the deviation pattern (SD or TD) from the standard sound stream was solely responsible for the results obtained. We used band-pass filtered click-trains that did not convey specific meanings to ensure that hemispheric lateralization for pre-attentive human auditory processing, represented by MMN(m), was not limited to the complex waveforms from natural sound sources (e.g. human voice or musical instruments), but in part originated from early, low-level auditory neural processing dealing with basic sound characteristics, namely, spectral and temporal features (Zatorre and Belin 2001; Tallal et al. 1993; Poeppel 2003; Boemio et al. 2005).

It seems plausible that spectral and temporal sound information is differentially encoded into neural activity (Bendor and Wang 2007; Sakai et al. 2009). Spectral information is encoded into the maximal movement position of the basilar membrane in the cochlea. Therefore, in case of the SD condition, the groups of inner hair cells corresponding to SD sounds were different from those corresponding to standard sounds. In contrast, TD sounds had similar frequency characteristics to standard sound signals. Similar groups of inner hair cells on the tonotopic map in the cochlea are activated. In order to detect the TD sound signal, the central auditory system should analyze the temporal patterns of neural activity. The present results demonstrated that the MMNm latencies elicited by TD were significantly longer than those elicited by SD (Figs. 3, 4). First, we have to consider the timing of the SD and TD onsets. Theoretically, SD is detectable from the first click of the TS in the cochlea, whereas TD detection requires the second click of the 60 Hz band-pass filtered click trains deviated from the standard 30 Hz band-pass filtered clicks or the missing second click of the standard 60 Hz band-pass filtered click trains during presentation of the deviant 30 Hz band-pass filtered click trains to manifest in the central auditory system. Therefore, we first subtracted 16.7 ms from the MMNm_TD latency in order to compare it with the MMNm_SD latency. Even after this adjustment, MMNm_TD was significantly longer than MMNm_SD (Fig. 4), which suggested that different neural mechanisms contribute to the detection of spectral and temporal sound deviants. Neural encoding of the temporal patterns of auditory signals took longer and appeared to take place at a higher level of the auditory system than spectral coding. Previous MEG studies (Okamoto et al. 2009, 2012) also support this hypothesis by demonstrating that the temporal changes elicited significantly delayed auditory N1m responses, with a major deflection in the auditory evoked response having a latency of approximately 100 ms (Näätänen and Picton 1987), than those elicited by spectral changes.

Auditory MMNm is a pre-attentive automatic brain response elicited by any change in auditory stimulation (Näätänen et al. 2007). In the present study, we used band-pass filtered click trains that did not convey specific meaning and subjects were distracted from the auditory modality; therefore, it is less likely that subjects involuntarily processed and perceived the test sounds as musical or speech signals. The obtained results indicated that the hemispheric asymmetry of auditory processing in humans starts from the basic, pre-attentive auditory processing level. Moreover, sound inputs were completely counter-balanced between the SD and TD conditions. Therefore, the hemispheric asymmetry of the MMNm responses elicited by the SD and TD could not be explained solely by stimulus features. The lateralized memory traces of basic auditory processes in terms of spectral and temporal sound features appear to be responsible for the results obtained. Recent human neuroimaging studies revealed that the functional hemispheric asymmetry of auditory processing was not limited to complex sound signals conveying specific meaning and rules (e.g. music and speech), but originated from the basic auditory processing level, namely, the temporal integration window (Poeppel 2003; Belin et al. 1998; Zatorre and Belin 2001; Zatorre et al. 2002). It is important to quickly and precisely encode environmental sounds in daily life. However, because of the trade-off between temporal and spectral analysis precision [Acoustic uncertainty principle; (Joos 1948; Zatorre et al. 2002)], it is impossible to achieve high spectral and high temporal sound analyses at the same time using one temporal integration window. A short temporal integration window leads to high temporal resolution, but relatively low spectral resolution of the sound analyses. On the other hand, a long temporal window leads to high spectral resolution, but relatively low temporal resolution of the sound analyses. Therefore, it seems plausible that the human auditory cortices in the left and right hemispheres adopt different integration time windows instead of applying one specific temporal integration time window in both hemispheres. Belin et al. (1998) and Poeppel (2003) hypothesized that the left hemisphere applied a shorter temporal integration window, resulting in a better temporal resolution capability, and the right hemisphere applied a longer temporal integration window, resulting in a better spectral resolution capability. In the present study, the longer temporal integration window with higher spectral resolution in the right hemisphere appears to have dominantly contributed to detecting spectrally deviated sound signals and resulted in relatively larger MMNm_SD amplitudes in the right hemisphere. In contrast, the shorter temporal integration window with high temporal resolution in the left hemisphere appears to have dominantly processed temporally deviated sound signals and resulted in relatively larger MMNm_TD amplitudes in the left hemisphere.

The MMNm amplitudes and latencies obtained in the sensor space and source space exhibited similar patterns (Figs. 3, 4): the MMNm_SD and MMNm_TD amplitudes were larger in the right and left hemispheres, respectively. However, the ANOVA examining MMNm amplitudes resulted in a significant interaction between DEVIANT_CONDITION and HEMISPHERE in the source space data [F_(1, 11)_ = 6.67, *p* < 0.03], but only a marginal trend toward significance was observed in the sensor space data [F_(1, 11)_ = 4.54, *p* = 0.056]. The main reason for this inconsistency may be that the neural sources in one hemisphere could influence the evoked magnetic fields in the contra-lateral magnetic sensors. Moreover, head sizes and head positions differed between subjects and the central sulcus of the subjects could shift from the center of the MEG dewar. Therefore, these factors may have led to a less robust statistical outcome in the RMS amplitudes of the MMNm responses than the MMNm source strengths.

In conclusion, using carefully constructed auditory stimuli that were counter-balanced between conditions and had clear time-locked onsets of SD and TD, the present study clearly demonstrated that neural processing dealing with spectrally deviated sounds were relatively dominant in the right hemisphere while those dealing with temporally deviated sounds were relatively dominant in the left hemisphere. These results strongly support the hypothesis that the human brain adopts asymmetric memory traces of basic spectral and temporal sound features in the left and right hemispheres in order to improve the detection of deviant sound signals.

## Electronic supplementary material

Below is the link to the electronic supplementary material.

Supplementary material 1 (MPEG 8 kb)

Supplementary material 2 (MPEG 8 kb)

Supplementary material 3 (MPEG 8 kb)

Supplementary material 4 (MPEG 8 kb)

## Abbreviations

ANOVA: Analysis of variance
MEG: Magnetoencephalography
MMN: Mismatch negativity
SD: Spectral deviant
TD: Temporal deviant
TS: Test stimulus
